# DATA ANALYTICS FOR HEALTH-RELEVANT EVENTS DETECTION BASED UPON LONGITUDINAL FITBIT HEART RATE DATA

**DOI:** 10.1093/geroni/igad104.2810

**Published:** 2023-12-21

**Authors:** Prathamesh Dharangutte, Jie Gao, Elinor Schoenfeld, Zongxing Xie, Yindong Hua, Fan Ye

**Affiliations:** Rutgers University, New Brunswick, New Jersey, United States; Rutgers University, PIscataway, New Jersey, United States; Stony Brook School of Medicine, Stony Brook, New York, United States; Stony Brook University, Stony Brook, New York, United States; Stony Brook University, Stony Brook, New York, United States; Stony Brook University, Stony Brook, New York, United States

## Abstract

The increasing prevalence of wearable devices enables low-cost, long-term collection of health relevant data such as heart rate, exercise, and sleep signals. Currently these data are used to monitor short term changes with limited interpretation of their relevance to health. These data provide an untapped resource to monitor daily and long-term activity patterns. Changes and trends identified from such data can provide insights and guidance to the management of many chronic conditions that change over time. In this study we conducted a machine learning based analysis of longitudinal heart rate data collected over multiple years from Fitbit devices. We built a multi-resolutional pipeline for time series analysis, using model-free clustering methods inspired by statistical conformal prediction framework. With this method, we were able to detect health relevant events, their interesting patterns (e.g., daily routines, seasonal differences, and anomalies), and correlations to acute and chronic changes in health conditions. We present the results, lessons, and insights learned, and how to address the challenge of lack of labels. The study confirms the value of long-term heart rate data for health monitoring and surveillance, as complementary to extensive yet intermittent examinations by health care providers.

